# The vaginal microbiota and innate immunity after local excisional treatment for cervical intraepithelial neoplasia

**DOI:** 10.1186/s13073-021-00977-w

**Published:** 2021-11-04

**Authors:** Anita Mitra, David A. MacIntyre, Maria Paraskevaidi, Anna-Barbara Moscicki, Vishakha Mahajan, Ann Smith, Yun S. Lee, Deirdre Lyons, Evangelos Paraskevaidis, Julian R. Marchesi, Phillip R. Bennett, Maria Kyrgiou

**Affiliations:** 1 Institute of Reproductive and Developmental Biology, Department of Metabolism, Digestion and Reproduction – Surgery and Cancer, Imperial College London, London, W12 0NN UK; 2 Department of Obstetrics & Gynaecology, Imperial College NHS Trust, London, W120HS UK; 3 Ronald Reagan UCLA Medical Center, UCLA Mattel Children’s Hospital, Santa Monica, CA USA; 4 Faculty of Health and Applied Sciences, University West of England, Bristol, Glenside Campus, Bristol, BS16 1DD UK; 5 Department of Obstetrics and Gynaecology, University Hospital of Ioannina, Ioannina, Greece; 6 Division of Digestive Diseases, Imperial College London, London, W2 1NY UK; 7 IRDB, Department of Gut, Metabolism and Reproduction - Surgery and Cancer, Imperial College London, Hammersmith Campus, 3rd Floor, Du Cane Road, London, W12 0NN UK

**Keywords:** Vaginal microbiota, Metataxonomics, Mucosal immunity, Cervical intraepithelial neoplasia, *Lactobacillus*

## Abstract

**Background:**

Vaginal microbiota (VMB) composition is altered in women with cervical intra-epithelial neoplasia (CIN) compared to healthy controls and is associated with disease progression. However, the impact of CIN excision on the VMB and innate immunity is not known. This observational study aims to explore the impact of CIN excision on the VMB, antimicrobial peptides (AMP) and proinflammatory cytokines.

**Methods:**

We sampled 103 non-pregnant, premenopausal women at the time of excisional treatment for CIN and at their 6-month follow-up visit. A further 39 untreated controls with normal cytology were also sampled. We used metataxonomics to group vaginal swab samples into community state types (CSTs) and ELISA to quantify cytokine and AMP levels in matched vaginal secretions. Analyses were performed to compare the bacterial composition and immune analyte levels before and after CIN excision and in healthy controls.

**Results:**

Women with CIN had significantly higher rates of *Lactobacillus* species depletion pre-treatment compared to healthy controls (CST IV 21/103, 20% vs 1/39, 3%, *p* = 0.0081). Excision did not change the VMB composition, with CST IV remaining significantly more prevalent after excision compared to untreated, healthy controls (CST IV 19/103, 20% vs 1/39, 3%, *p* = 0.0142). *Prevotella bivia* and *Sneathia amnii* were significantly higher in samples before treatment compared to untreated controls, and *Prevotella bivia* remained significantly higher amongst the treated, with less *Lactobacillus crispatus* compared to untreated controls. IL-1β and IL-8 remained significantly elevated pre- (*p* < 0.0001 and *p* = 0.0014, respectively) and post-treatment (*p* < 0.0001 and *p* = 0.0035, respectively) compared to untreated controls. Levels of human beta-defensin-1 and secretory leukocyte protease inhibitor were both significantly reduced following CIN excision (*p* < 0.0001); however, their levels remained lower than controls post-treatment.

**Conclusions:**

Women with CIN have an increased prevalence of *Lactobacillus* sp. depletion, high-diversity VMB composition, and higher levels of proinflammatory cytokines and AMPs compared to normal controls. Surgical excision of the disease reduces levels of vaginal AMPs but does not alter VMB composition or cytokine levels. These findings suggest that women with CIN have an inherent predisposition to a high-diversity proinflammatory environment that is not corrected by disease excision. The failure to re-establish a *Lactobacillus*-enriched CST may explain why women remain at high risk of pre-invasive and invasive disease recurrence.

**Supplementary Information:**

The online version contains supplementary material available at 10.1186/s13073-021-00977-w.

## Background

Persistent infection with oncogenic high-risk human papillomavirus (HPV) subtypes is necessary for the development of cervical cancer and its precursor cervical intra-epithelial neoplasia (CIN) [1]. Infection with HPV is the norm rather than the exception, although most women are able to clear the virus through an incompletely understood immune response [2]. Only a fraction of women develop persistence and ultimately progressive CIN. A number of host, viral genetic and epigenetic factors have been implicated and are currently under investigation, although growing evidence has led to the hypothesis that the vaginal microbiota (VMB) plays a role in the outcome of this virally induced disease process [3–9].

The vagina is typically populated by *Lactobacillus* species creating an acidic environment rich in bioactive compounds that is protective against ascending infections and overgrowth of pathobionts [10, 11]. Numerous cross-sectional observational studies have reported an association between depletion of *Lactobacillus* species (spp.) and greater bacterial diversity to the presence of HPV, CIN and invasive cancer [4, 8, 12–16]. Although reporting of these associations is of value, these do not permit further exploration as to whether these VMB alterations are a cause or consequence of the virus and/or the disease, or are due to inherent genetic predisposition or environmental impact on colonisation.

A handful of longitudinal cohort studies have more recently attempted to correlate HPV and CIN persistence and clearance to the VMB in an attempt to explore further a plausible causal relationship. Brotman and colleagues reported in a small cohort of 32 women serially sampled over a 16-week period [17] that community state type IV (CST IV), associated with high diversity and *Lactobacillus* sp. depletion, and was associated with a higher chance of HPV acquisition and persistence. More recently, a longitudinal cohort of 87 women sampled serially every 3 months and followed up for 24 months reported for the first time that *Lactobacillus* sp. depletion is associated with higher rates of persistent/progressive CIN2 at 12- and 24-month follow-up, whilst *Lactobacillus* sp. dominance associated with increased rates of regression [6].

Any attempt to infer further on causality requires interventional studies that will explore how excision of the disease, i.e. CIN, will alter the vaginal microbiota and vaginal microenvironment. Findings that would be consistent with VMB reversal to *Lactobacillus* sp. dominance after CIN excision would imply that the virus and precancer are the drivers of VMB alteration to high diversity. Conversely, an unaltered microbiota despite the removal and absence of disease and infection would be suggestive of inherent genetic, epigenetic and environmental factors that determine the vaginal microbiota that are likely to make these women more susceptible to the acquisition and persistence of HPV and promote further disease progression. Currently, there are no such studies.

In an attempt to move beyond association, studies are beginning to explore the mechanisms underpinning the impact of the VMB on cervical disease. Most of these suggest that *Lactobacillus* spp. depleted, high diversity VMB alters the mucosal immune environment, predisposes the development of a proinflammatory *milieu* with over-expression of pro-carcinogenic biomarkers and cyto/chemokines, which in turn promotes carcinogenesis [8, 9, 18–20]. Previous studies have reported higher proinflammatory cytokines, such as IL-1β and IL-8 in women with CIN [18], and higher TNF-α levels are associated with HPV persistence [19]. In contrast, suppression of the antiviral cytokine INFγ in high-grade disease has been proposed to promote disease progression [21]. Antimicrobial peptides (AMPs) are important mediators of innate mucosal immunity against both bacterial and viral pathogens with human beta-defensin-1 (hBD1) and secretory leucocyte protease inhibitor (SLPI) being both constitutively and inducibly expressed by the cervix [22, 23]. Polymorphisms in the DEFB1 gene that encodes hBD-1 have been associated with increased susceptibility to HPV infection [24], whilst SLPI is known to have potent anti-viral activity against HPV in head and neck cancers [25] and is upregulated in CIN [26] and cervical cancer [27]. Although the role of both cytokine and AMPs in cervical disease has been previously explored, the interplay between the VMB, HPV and inflammation are incompletely understood.

This prospective observational study of women planned for local cervical treatment aims to explore the impact of the excision of disease on the vaginal microbiota composition, innate immune and inflammatory response and further explore how these pre- and post-treatments compare to healthy untreated controls.

## Methods

### Study population—inclusion and exclusion criteria

We included 103 pre-menopausal, non-pregnant women aged 18 to 45, planned for local excisional treatment for CIN attending the colposcopy clinics at Imperial College NHS Healthcare Trust. Samples were collected prior to treatment and 6 months post-treatment at the follow-up visit. Swabs were also collected at a single time point from a control population of 39 women with normal cytology. Women were included irrespective of their HPV status (treatment and control groups), ethnicity, parity, smoking habits, phase in their menstrual cycle and use of contraception. Women who were HIV- or hepatitis B/C-positive, with autoimmune disorders, who received antibiotics or pessaries within 14 days of sampling or had a previous history of cervical treatment were excluded. Patients were anonymised and assigned a unique digital identifier. Detailed medical and gynaecological history was collected including the type of contraception, last menstrual period, time since last sexual intercourse and douching practices. We also collected data on cytology, HPV test and histology. Cytology was classified as normal, borderline or mild dyskaryosis (low-grade squamous intraepithelial lesion [LSIL]) and moderate or severe dyskaryosis (high-grade squamous intraepithelial lesions [HSIL]). The histology groups included normal, CIN1, CIN2 and CIN3. Ethnicity was self-reported as Caucasian, Asian or Black. All patients gave informed written consent.

### Sample collection and processing

Samples were collected using a sterile speculum without lubricant immediately prior to colposcopy or collection of any other clinically indicated samples to avoid contamination. A BBL™ CultureSwab™ containing liquid Amies with a rayon tip (Becton Dickinson, Oxford, UK) was used to collect samples for 16S rRNA gene sequencing, and a rayon swab (Medical Wire & Equipment) inserted into phosphate-buffered saline–containing protease inhibitor cocktail (Sigma, Dorset, UK) was used for enzyme-linked immunosorbent assay experiments. Samples were immediately transported on ice and stored at − 80 °C until analysis. Whole genomic bacterial DNA was extracted from the liquid Amies swab solution using a QIAmp DNA mini kit (Qiagen, Venlo, The Netherlands) as previously described [4].

### Measurement of dimensions of excision

Transvaginal ultrasonography (TVUS) was performed immediately prior to cervical excision using a Voluson E6 with a 5–9 MHz (RIC5-9-RS series) transvaginal probe (GE Healthcare, Zipf, Austria) to assess the cervical length. We used an electronic digital calliper (Maplin Electronics, Rotherham, UK) to measure the dimensions of the cone removed prior to fixation. The proportion of length excised was calculated. Repeat cervical length ultrasound at 6 months was performed to assess length regeneration. Details on the principles have been previously described [28–30].

### Illumina MiSeq sequencing of 16S rRNA gene amplicons and sequence analysis

The V1–V3 hypervariable regions of 16S rRNA genes were amplified, sequenced and analysed as previously described [31] using a forward primer constructed with the Illumina i5 adapter (5′-AATGATACGGCGACCACCGAGATCTACAC-3′), an 8-base pair (bp) bar code, a primer pad (forward, 5′-TATGGTAATT-3′), and the 28F primer (5′-GAGTTTGATCNTGGCTCAG-3′) and the reverse fusion primer with the Illumina i7 adapter (5′-CAAGCAGAAGACGGCATACGAGAT-3′), an 8-bp bar code, a primer pad (reverse, 5′-AGTCAGTCAG-3′), and the 519R primer (5′-GTNTTACNGCGGCKGCTG-3′). Mothur (version 1.35.1) was used to define OTU taxonomies using the ribosomal database project (RDP, Release 11) MultiClassifier script to generate the RDP taxonomy from phylum to genus level [32], and the USEARCH algorithm combined with the cultured representatives from the RDP [33] and STIRRUPS databases [34] for species allocation. Sequencing was conducted at Research and Testing Laboratory (Lubbock, TX, USA).

### Cytokines and anti-microbial peptides

Cytokine levels (IL-1β, IL-2, IL-4, IL-6, IL-8, IL-10, IFN-γ, MIP-1α, RANTES and TNF-α) in cervicovaginal secretions were determined using the Magnetic Luminex Screening Assay multiplex kit (R&D Systems, Minneapolis, MN, USA). hBD-1 levels were determined using a Human BD-1 ELISA Development Kit (Peprotech, London, UK) and human SLPI levels with the ELISA kit (Hycult Biotech, Uden, The Netherlands). For the purpose of analysis, cytokine and AMP levels were normalised to the total protein content of the sample determined using the *DC* Protein Assay Kit (Bio-Rad, Hemel Hempstead, UK) in order to reduce inter-individual variability of immune marker measurement [35]. All kits were used according to the manufacturer’s instructions and analysed in duplicate on the BioPlex 100 Analyzer (Bio-Rad). The lower limit of quantification (LLOQ) was defined as the lowest point on the standard curve for each individual analyte.

To further explore how the disease affects the AMP expression within the cervix, we used immunohistochemistry (IHC) to compare relative staining in normal versus diseased tissue within the same section. To further explore the impact of excision and scarring on AMP expression, we compared the sections from the first versus the second cone in two women requiring repeat excision. Slides were cut from paraffin-embedded blocks and deparaffinised, and a horseradish peroxidase-3,3′ Diaminobenzidine Cell & Tissue staining kit (R&D systems) was used according to the manufacturer’s instructions along with hBD-1 (Peprotech) at 1:10 dilution and SLPI antibodies (Hycult Biotech) used at 1:5 dilution. Meyer’s haematoxylin was used to counterstain the slides.

### HPV genotyping

HPV DNA testing and genotyping were performed using Abbott RealTime High Risk (HR) HPV assay on Abbott M2000 platform according to the manufacturer’s guidelines.

### Data synthesis and analysis

Analysis of vaginal microbiota composition data using the Statistical Analysis of Metagenomic Profiles (STAMP) package (v.2.1.3) [36]. Multivariate analysis using hierarchical clustering analysis (HCA) by centroid clustering was performed with a density threshold of 0.75. Classification at species level was according to the previously described vaginal community state types (CSTs) I–V [37]. Linear discriminant analysis (LDA) effect size (LEfSe) analysis was used to identify taxa significantly overrepresented according to clinical outcome, through all taxonomic levels [38] using taxonomic relative abundance, with per-sample normalisation and default settings for alpha values (0.05) for the factorial Kruskal-Wallis test amongst classes and pairwise Wilcoxon test between subclasses. A logarithmic LDA score greater than 2 was used to determine the discriminative features.

**Fig. 1:**
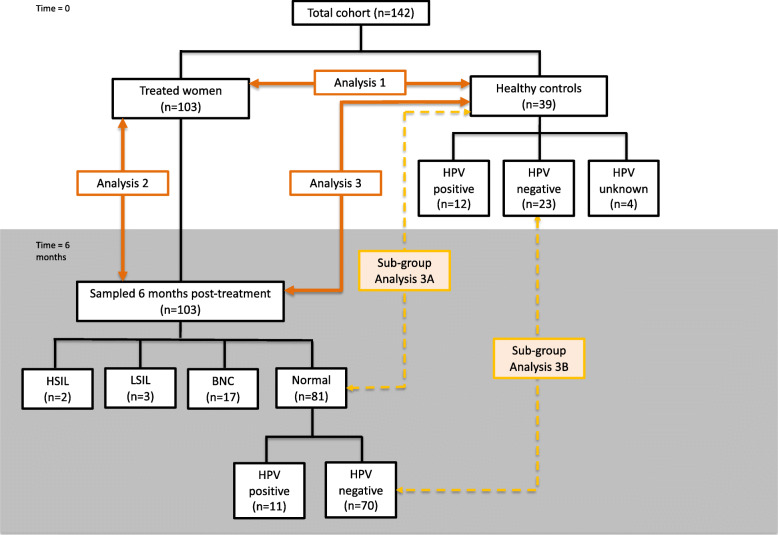
Study groups and analyses. Analysis 1: Comparisons of 103 women immediately prior to excisional treatment versus 39 healthy, untreated controls with normal cytology. Analysis 2: Comparisons of 103 paired samples of women immediately prior to excisional treatment versus 6 months after. Analysis 3: Comparisons of 103 treated women at 6-month follow-up versus 39 healthy, untreated controls with normal cytology. Subgroup analysis 3A: Comparison on 81 treated women with normal cytology at follow-up versus 39 healthy, untreated controls with normal cytology. Subgroup analysis 3B: Comparison of 70 treated women with negative cytology and HPV test versus 23 healthy, untreated controls with negative cytology and HPV test. BNC, borderline nuclear changes; HSIL high-grade squamous intraepithelial lesion; HPV, human papillomavirus

We performed a series of analyses of the vaginal microbiota composition and antimicrobial peptide (AMP) and cytokine expression in order to understand the impact of treatment on the vaginal microbiota and inflammatory response. We aimed to explore the following: (A) how these at baseline just before treatment (*n* = 103) compared to healthy, untreated controls (*n* = 39) (analysis 1); (B) how these are altered pre- and post-excisional treatment (*n* = 103 paired samples) (analysis 2); and (C) how this post-treatment (*n* = 103) compare to healthy untreated controls (*n* = 39) (analysis 3). We performed analysis 3 to assess whether any observed differences could be attributed to the treatment itself or rather due to the removal of the disease (CIN). Within these analyses, we performed further subgroup analyses comparing women post-treatment with normal cytology (*n* = 81) to controls (*n* = 39) (analysis 3A) and women post-treatment with negative HPV tests and cytology (*n* = 70) to HPV-negative controls (*n* = 23) (analysis 3B) (Fig. 1).

As oncological and reproductive outcomes [39–41] have been found to correlate with the cone length and cervical regeneration, we compared VMB, AMPs and cytokine dynamics by comparing the upper versus the lower 50th percentile from the median for the absolute cone depth (mm), the proportion (percentage) of cervical length excised and the proportion of length regeneration.

Fisher’s exact test, chi-squared test, Mann–Whitney *U* tests and *t* tests were performed where appropriate using (GraphPad Prism 8, [GraphPad Software Inc., La Jolla, CA, USA]). Pearson’s correlation coefficient was used to examine the associations between bacterial taxa, AMPs and cytokines. A *p* value less than 0.05 was considered statistically significant.

## Results

### Patient cohort and characteristics

We recruited 106 women undergoing excisional treatment; three became pregnant after treatment and before the 6 months follow-up visit and were excluded from further analysis. We recruited a further 39 healthy controls with normal cytology.

The patient characteristics across all analyses are detailed in Table 1 and Additional file 1: Table S1. The average time to follow-up was 6 months (range 5–12 months). The control group were found to be younger than the treated women at the time of follow-up (*p* = 0.013). Other characteristics were not significantly different between the compared groups. There was an equal distribution of samples collected in the follicular or luteal phase of the cycle and in the rate of women that had intercourse within 48 h from sample collection between all the three groups. Two women were retreated due to the presence of high-grade disease at 6 months. The majority were treated for high-grade CIN (CIN1 15/103, 15%; CIN2 40/103, 39%); CIN3 48/103, 46%).

**Table 1:** Patient characteristics

	Analysis 1			Analysis 2			Analysis 3		
	Pre-treatment, ***n*** = 103	Control, ***n*** = 39	***p*** value	Pre-treatment, ***n*** = 103	Post-treatment, ***n*** = 103	***p*** value	Post-treatment, ***n*** = 103	Control, ***n*** = 39	***p*** value
**Age, years**			0.0573			0.4131			**0.0130**
Mean (SD, range)	31.9 (5.2, 25–45)	30.1 (4.4, 20–43)		31.9 (5.2, 25–45)	32.5 (5.3, 25–46)		32.5 (5.3, 25–46)	30.1 (4.4, 20–43)	
**Ethnicity,** ***n*/*N*** **(%)**			0.0659			> 0.9999			0.0659
Caucasian	87/103 (85)	30/39 (77)		87/103 (85)	87/103 (85)		87/103 (85)	30/39 (77)	
Asian	10/103 (10)	2/39 (5)		10/103 (10)	10/103 (10)		10/103 (10)	2/39 (5)	
Black	6/103 (5)	7/39 (18)		6/103 (5)	6/103 (5)		6/103 (5)	7/39 (18)	
**Parity,** ***n*/*N*** **(%)**			0.5329			> 0.9999			0.5329
Nulliparous	73/103 (71)	30/39 (76)		73/103 (71)	73/103 (71)		73/103 (71)	30/39 (76)	
Parous	30/103 (29)	9/39 (24)		30/103 (29)	30/103 (29)		30/103 (29)	9/39 (24)	
**Smoking status,** ***n*/*N*** **(%)**			0.1909			> 0.9999			0.2647
Current smoker	27/103 (26)	6/39 (15)		27/103 (26)	26/103 (25)		26/103 (25)	6/39 (15)	
Non-smoker	76/103 (74)	33/39 (85)		76/103 (74)	77/103 (75)		77/103 (75)	33/39 (85)	
**Phase of menstrual cycle,** ***n*/*N*** **(%)**			0.6472			0.4655			0.5388
Luteal	55/103 (53)	23/39 (58)		55/103 (53)	50/103 (48)		50/103 (48)	23/39 (58)	
Follicular	38/103 (37)	14/39 (36)		38/103 (37)	46/103 (46)		46/103 (46)	14/39 (36)	
Unknown	10/103 (10)	2/39 (5)		10/103 (10)	7/103 (7)		7/103 (7)	2/39 (5)	
**Contraception,** ***n*/*N*** **(%)**			0.7316			0.9982			0.6958
Nil	35/103 (34)	19/39 (49)		35/103 (34)	34/103 (33)		34/103 (33)	19/39 (49)	
Condoms	17/103 (16)	5/39 (13)		17/103 (16)	18/103 (17)		18/103 (17)	5/39 (13)	
COCP	37/103 (36)	12/39 (31)		37/103 (36)	37/103 (36)		37/103 (36)	12/39 (31)	
POP	5/103 (5)	1/39 (2)		5/103 (5)	5/103 (5)		5/103 (5)	1/39 (2)	
Copper IUD	2/103 (2)	0/39 (0)		2/103 (2)	1/103 (1)		1/103 (1)	0/39 (0)	
Mirena IUS	5/103 (5)	1/39 (2)		5/103 (5)	6/103 (6)		6/103 (6)	1/39 (2)	
Contraceptive implant	2/103 (2)	1/39 (2)		2/103 (2)	2/103 (2)		2/103 (2)	1/39 (2)	
**Time since last intercourse,** ***n*/*N*** **(%)**			0.7684			> 0.9999			0.7684
> 48 h	92/103 (89)	34/39 (87)		92/103 (89)	92/103 (89)		92/103 (89)	34/39 (87)	
< 48 h	11/103 (11)	5/39 (13)		11/103 (11)	11/103 (11)		11/103 (11)	5/39 (13)	
**HPV status**			–	–		–	–		–
Negative	–	–		–	–		82/103 (80)	23/39 (59)	
Positive	–	–		–	–		21/103 (20)	12/39 (31)	
Unknown	–	–		–	–		0/103 (0)	4/39 (10)	
**Cytology**									
Negative	–	–		–	–		81/103 (79)	39/39 (100)	
Borderline nuclear changes	–	–		–	–		17/103 (16)	0/39 (0)	
LSIL	–	–		–	–		3/103 (3)	0/39 (0)	
HSIL	–	–		–	–		2/103 (2)	0/39 (0)	
**Cytology and HPV status**			–	–		–	–		–
Normal, HPV-ve	–	–		–	–		70/103 (69)	23/39 (59)	
Normal, HPV+ve	–	–		–	–		11/103 (10)	12/39 (31)	
Normal, HPV status unknown	–	–		–	–		0/103 (0)	4/39 (10)	
BNC, HPV-ve	–	–		–	12/103 (11)		12/103 (11)	0/39 (0)	
BNC, HPV+ve	–	–		–	5/103 (5)		5/103 (5)	0/39 (0)	
LSIL, HPV-ve	–	–		–	0/103 (0)		0/103 (0)	0/39 (0)	
LSIL, HPV+ve	–	–		–	3/103 (3)		3/103 (3)	0/39 (0)	
HSIL	–	–		–	2/103 (2)		2/103 (2)	0/39 (0)	

*BNC* borderline nuclear changes, *CIN* cervical intraepithelial neoplasia, *COCP* combined oral contraceptive pill, *HSIL* high-grade squamous intraepithelial neoplasia, *HPV* human papillomavirus, *IUD* intrauterine device, *IUS* intrauterine system, *LSIL* low-grade squamous intraepithelial neoplasia, *POP* progesterone-only pill, *SD* standard deviation

In total, 2,977,082 reads were obtained from 245 samples with an average number of reads per sample of 11,400 and the mean and median read lengths of 513 and 520 bp, respectively. To avoid sequencing bias, operational taxonomic units (OTUs) were sub-sampled to the lowest read count of 296, which retained 99.7% of OTU counts and still provided coverage of > 97.9% for all samples. Following the removal of singletons and rare OTUs, a total of 77 taxa were identified in the vaginal microbiota of the study cohort.

**Fig. 2:**
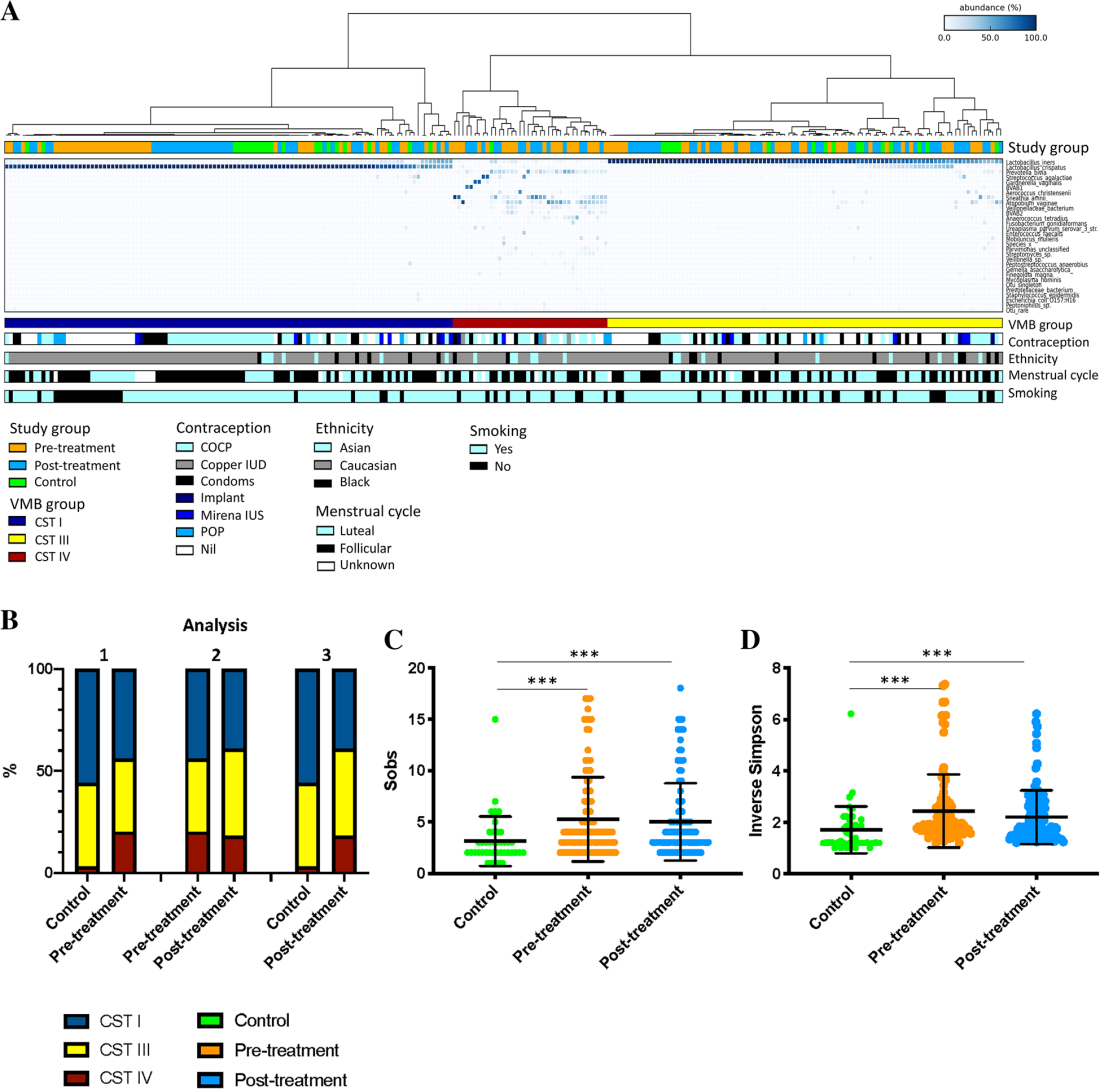
Heatmap showing the vaginal microbiota composition in 103 women before (orange) and after (blue) excisional treatment and in 39 healthy, untreated controls (green) (**A**) and changes in richness (**C**) and diversity (**D**) indices associated with disease status. **A** Three of the previously described community state types (CSTs) were identified in our cohort. **B** CST IV was significantly more prevalent in the treatment cohort both before and after treatment compared to 39 untreated controls (*p* = 0.0081 and 0.0142, respectively, chi-squared test). There was no significant change in the vaginal microbiome composition before and after treatment. **C** Richness was significantly greater in women planned for excision both before (*p* = 0.0003, unpaired *t* test) and after (*p* = 0.0005, unpaired *t* test) treatment when compared to healthy untreated controls. **D** Diversity was also increased in women planned for treatment before (*p* < 0.0001, unpaired *t* test) and after (*p* = 0.0005 unpaired *t* test) treatment compared to controls. Richness or diversity was no different before and after treatment. CST, community state type; Sobs, species observed; VMB, vaginal microbiota. Dots depict the individual samples; error bars denote mean ± standard deviation, ***p < 0.001; **p < 0.01; *p < 0.05)

Three of the five CSTs previously described by Ravel and colleagues [37] were observed in the study cohort, CST I (*L. crispatus*), III (*L. iners*) and IV (high-diversity, *Lactobacillus-*deplete) (Fig. 2a).

### The vaginal microbiota composition

#### Pre-treatment versus normal controls—analysis 1

**Fig. 3:**
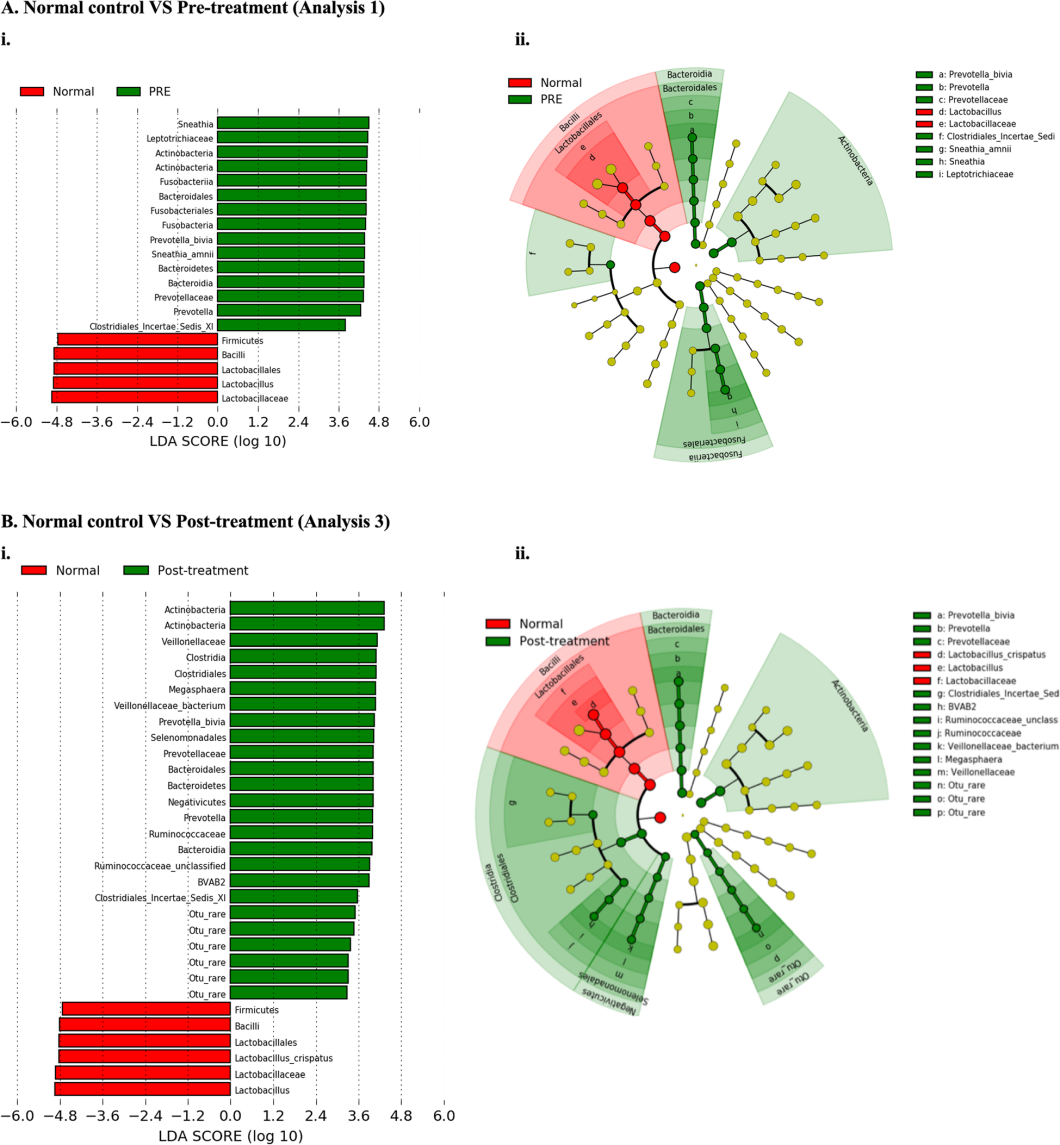
LEfSe analysis identified vaginal microbiota differentially abundant taxa in the comparison of women pre-treatment (analysis 1) and post-treatment (analysis 3) versus normal untreated controls. **A** Analysis 1: *Prevotella bivia* and *Sneathia amnii* were significant represented at a species level in women prior to treatment compared to untreated controls. **B** Analysis 3: *Prevotella bivia* remained to be significantly overrepresented post-treatment, along with BVAB2 compared to controls, who had significantly higher levels of *Lactobacillus crispatus.* There were no differentially abundant taxa for the comparison before and after treatment, and therefore, neither a histogram nor cladogram can be generated (analysis 2). Histogram of LDA scores was found to differ significantly in abundance between women who had normal cytology pre-treatment (analysis 1) or post-treatment (analysis 3) compared to normal, untreated controls. (i) Cladogram representing the taxa at all phylogenetic levels with significantly different abundance in the compared two groups. The size of the circle is proportional to the abundance of taxon represented. LDA, linear discriminant analysis. NB. Analysis restricted to top 20 taxa with all remaining taxa denoted as ‘other’

A significantly greater rate of CST IV VMB composition was observed in our cohort of 103 women undergoing excisional treatment for CIN when compared to 39 healthy untreated controls (pre-treatment CST IV; 21/103, 20% vs controls; 1/39, 3%; *p* = 0.0081, chi-squared test). There was no significant difference between the rates of CST I (pre-treatment CST I; 45/103, 44% vs controls; 22/39, 56%; *p* = 0.1920, chi-squared test) or CST III (pre-treatment CST III; 37/103, 36% vs controls; 16/39, 41%; *p* = 0.6978, chi-squared test) (Fig. 2b, Table 2). Consistent with increased rates of CST IV in the pre-treatment cohort compared to controls, vaginal microbiota richness as measured by the number of species observed and diversity, represented by the inverse Simpson index, was also found to be higher in women pre-treatment (*p* = 0.0003 and *p* < 0.0001, respectively, chi-squared test) (Fig. 2c, d). LEfSe analysis demonstrated a significantly increased abundance of *Prevotella bivia* and *Sneathia amnii* in women prior to treatment compared to controls, who had significantly greater levels of the *Lactobacillus* genus (Fig. 3a).

**Table 2:** Vaginal microbiota composition according to disease state

	CST I (*L. crispatus*)	CST III (*L. iners*)	CST IV (*Lactobacillus* sp. deplete, high diversity)
**Analysis 1**			
Pre-treatment, *n* = 103	45/103 (44)	37/103 (36)	21/103 (20)
Control, *n* = 39	22/39 (56)	16/39 (41)	1/39 (3)
*p* value	0.1920	0.6978	**0.0081**
**Analysis 2**			
Pre-treatment, *n* = 103	45/103 (44)	37/103 (36)	21/103 (20)
Post-treatment, *n* = 103	40/103 (39)	44/103 (43)	19/103 (18)
*p* value	0.5715	0.3922	0.8604
**Analysis 3**			
Post-treatment, *n* = 103	40/103 (39)	44/103 (43)	19/103 (18)
Control, *n* = 39	22/39 (56)	16/39 (41)	1/39 (3)
*p* value	0.0873	1.000	**0.0142**
**Sub-group analysis 3A**			
Post-treatment, normal cytology, *n* = 81	32/81 (40)	35/81 (43)	14/81 (17)
Control, *n* = 39	22/39 (56)	16/39 (41)	1/39 (3)
*p* value	0.1166	0.8463	**0.0354**
**Sub-group analysis 3B**			
Post-treatment, normal cytology, HPV-negative, *n* = 70	27/70 (38)	30/70 (43)	13/70 (19)
Control, HPV negative, *n* = 23	12/23 (52)	10/23 (44)	1/23 (4)
*p* value	0.3309	1.0000	0.1758

*p* values represented are two-tailed Fisher’s exact (if < 5 observations in any group) and *χ*^2^ tests (where > 5 observations in every group)

*CST* community state type, *L. Lactobacillus*, *HPV* human papillomavirus

#### Pre- versus post-treatment—analysis 2

**Fig. 4:**
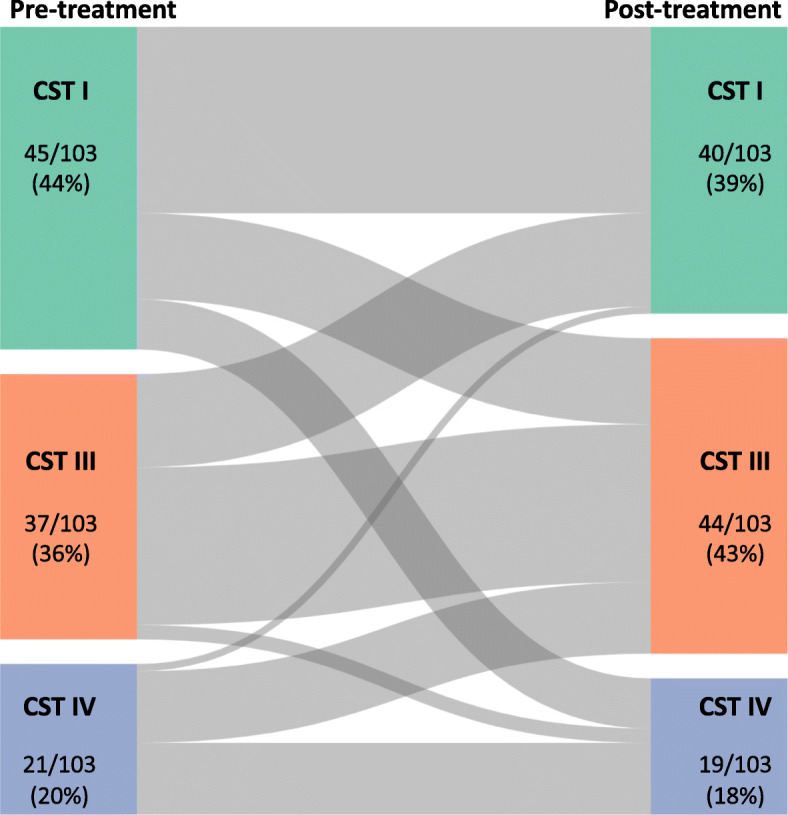
Sankey plot showing dynamics of vaginal microbiota composition pre- and post-treatment. Most patients (58/103, 56%) kept a stable vaginal microbiota CST before and after treatment. The majority of the 45 women who switched to another CST, switched between *Lactobacillus* sp.-dominant states (25/45, 56%)

Excisional treatment did not have any significant impact on the VMB composition when comparing paired samples from 103 women on the day of treatment and at 6-month follow-up. Proportions of each of the three CSTs were not significantly different at the two time points (CST I, *p* = 0.5715; CST III, *p* = 0.3922; CST IV, *p* = 0.8604, chi-squared test) (Fig. 2, Table 2) There was no difference between richness or diversity between pre- and post-treatment samples in the 103 matched women (Fig. 2c, d). LEfSe analysis did not detect any differentially abundant taxa between the two groups. The dynamics of the distribution of CST’s pre- and 6-months post-treatment are shown in a Sankey plot (Fig. 4). Of the 103 treated patients, 58 (56%) had the same vaginal microbiota CST on the day of treatment and at their 6-month follow-up appointment. Women with CST III were most likely to have the same CST before and after treatment (22/37, 59%), followed by CST I (26/45, 58%) and finally CST IV (10/21, 48%). Of the 45 women who switched to another CST, the majority switched between *Lactobacillus* sp.-dominant states (25/45, 56%). Nine women switched from a *Lactobacillus* sp.-dominant CST to CST IV (9/45, 20%), and 11 changed in the opposite direction from CST IV to a *Lactobacillus* spp.-dominant CST (11/45, 24%).

There was no evidence to suggest that more extensive excisions with larger cone lengths affected the vaginal microbiota. The CST distribution of rates and dynamics was similar when comparing the upper versus the lower 50th percentile for absolute or proportional length excised and proportional cervical length regeneration (Additional **f**ile 1: Table S2). The mean absolute and proportional cone length and cervix were similar for all CSTs (Additional **f**ile 1: Table S3). We performed the same analysis for absolute and proportional volume excised and found no difference (data not shown).

#### Post-treatment versus normal controls—analysis 3

Compared to controls, CST IV type VMB remained more prevalent in women post-CIN excision at the 6-month follow up (post-treatment CST IV 19/103, 18% vs controls 1/39, 3%; *p* = 0.0142, chi-squared test). There was no difference in the rates of CST III (44/103, 43% vs controls 16/39, 41%, *p* = 1.000, chi-squared test), and there was a trend for slightly higher rates of CST I in controls which was approaching significance (post-treatment CST I 40/103, 39% vs controls 22/39, 56%; *p* = 0.0873, chi-squared test) (Fig. 2, Table 2). Similarly, richness and diversity remained significantly higher post-treatment compared to controls (*p* = 0.0003 and *p* < 0.0001, respectively, unpaired *t* test) (Fig. 2c, d). LEfSe analysis showed that BVAB2, *Prevotella bivia* and *Veillonelaceae* OTU were significantly overrepresented in the treated cohort compared to controls who had greater levels of *Lactobacillus crispatus* present (Fig. 3b). *Sneathia amnii* was no longer overrepresented in the post-treatment group compared to controls (analysis 3), as it was when pre-treatment samples were compared to normal controls (analysis 1), and when we compared the mean relative abundance of this bacteria in the pre- and post-treatment (Additional file 1: Figure S2).

To explore whether it is the disease or inherent factors that drive changes in VMB composition, we restricted our subgroup analyses to post-treatment women with negative cytology (subgroup 3A) and negative HPV and cytology (subgroup 3B) (Table 2, Additional file 1: Table S1, Figure S3). For sub-analysis 3A, we removed 22 treated women with abnormal cytology, leaving 81 treated women versus 39 normal cytology controls. The rate of CST IV after treatment compared to controls remained to be significantly higher (post-treatment CST IV 14/81, 17% vs controls 1/39, 3%; *p* = 0.0354, chi-squared test) (Table 2). CST I and III rates were not significantly different between the two groups (*p* = 0.1166 and *p* = 0.8463, respectively, chi-squared test). LEfSe analysis again found *Lactobacillus crispatus* to be significantly enriched in untreated controls compared to the treated group, and the same three species: BVAB2, *Prevotella bivia* and *Veillonelaceae* OTU, all known to be associated with a high-diversity bacterial vaginosis-type VMB composition were significantly enriched in treated women (Additional file 1: Figure S1A). For sub-analysis 3B, we removed 33 women leaving 70 women with negative HPV and cytology and 16 HPV-positive controls leaving 23 HPV and cytology negative controls. In treated women, the rate of CST IV remained almost four times that of healthy controls (post-treatment CST IV 13/70, 19% vs controls 1/23, 4%; *p* = 0.1758, chi-squared test), but was no longer significant, likely due to small sample size. Relative abundance of *Lactobacillus crispatus* remained higher in samples from untreated controls compared to treated women according to LEfSe analysis, with the latter enriched for *Atopobium vaginae* and a *Veillonelaceae* OTU (Additional file 1: Figure S1B).

### hBD1 and SLPI levels

**Fig. 5:**
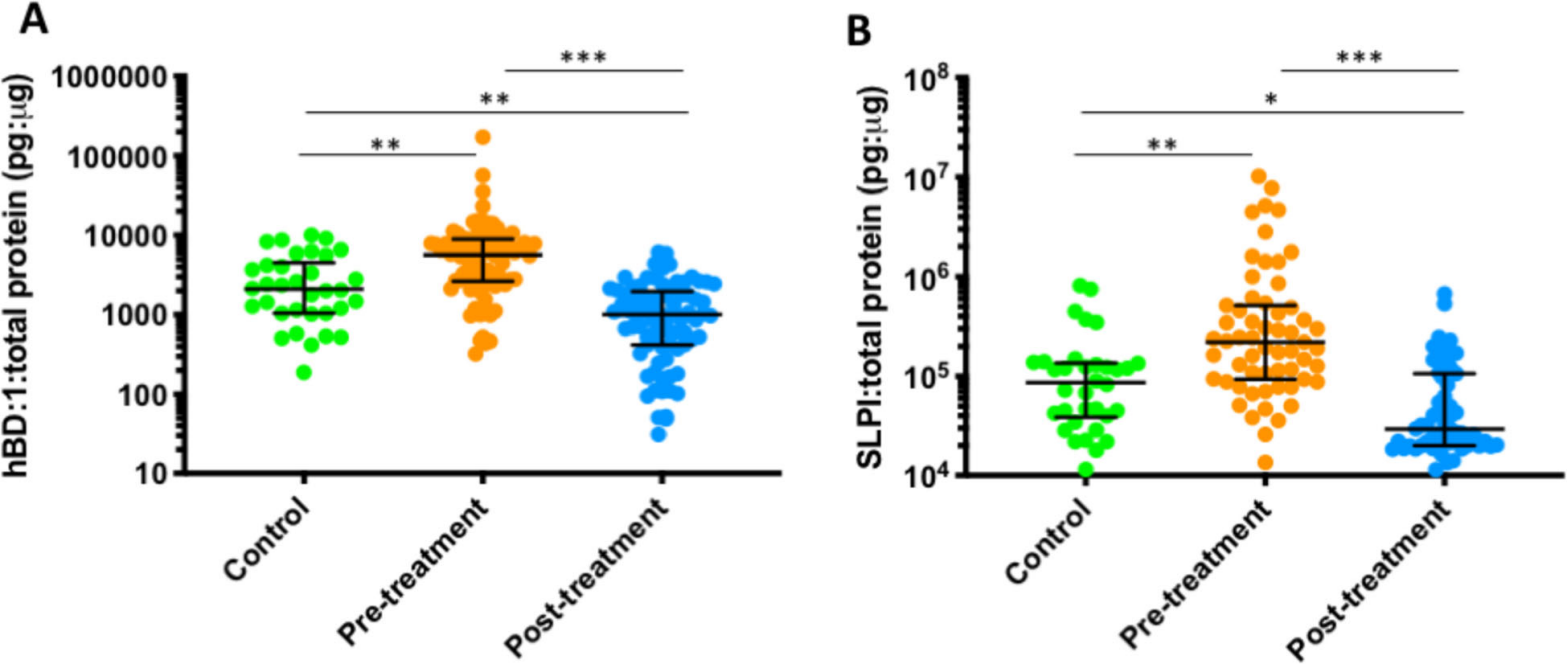
Levels of hBD-1 (**A**) and SLPI levels (**B**) for 80 treated and 34 untreated healthy controls. Levels prior to treatment were significantly higher than the controls (hBD-1 *p* = 0.0033, SLPI *p* = 0.0006, unpaired *t* test) (analysis 1). Excisional treatment led to a significant reduction of levels for both hBD-1 and SLPI when assessed in paired samples (hBD-1 *p* < 0,0001, SLPI *p* < 0.0001, paired *t* test) (analysis 2). The levels post-treatment were significantly lower than those of controls (hBD-1 *p* < 0.0029, SLPI *p* = 0.0382, unpaired *t* test) (analysis 3). Dots depict individual samples; error bars denote median and interquartile ranges, ***p < 0.001; **p < 0.01; *p < 0.05

The levels of hBD1 and SLPI were normalised to total protein levels in 80 treated women and 34 controls (Additional file 1: Table S4, Fig. 5). Levels for both peptides were higher prior to treatment compared to healthy controls (hBD-1, *p* = 0.0033; SLPI, *p* = 0.0006; unpaired *t* test, analysis 1). The paired samples pre- and post-treatment showed a significant reduction for both (hBD-1, *p* < 0.0001; SLPI, *p* < 0.0001; paired *t* test, analysis 2). The levels fell significantly after treatment and were significantly lower than healthy controls (hBD-1, *p* < 0.0029; SLPI, *p* = 0.0382; unpaired *t* test, analysis 3). In the comparison of only post-treatment cytology negative women (*n* = 65), AMP levels were significantly less in treated women compared to the healthy controls (*n* = 34) (hBD-1 *p* = 0.0003 and SLPI *p* = 0.0009; unpaired *t* test, subgroup analysis 3A), In the comparison of HPV and cytology-negative women (*n* = 50) to HPV-negative cytology controls (*n* = 20), the same observations were seen (hBD-1 *p* = 0.0006 and SLPI *p* = 0.0016; unpaired *t* test, subgroup analysis 3B). There was no correlation between the levels of hBD1 or SLPI and change in cervical length and volume or according to the proportion of length or volume excision (data not shown). Immunohistochemical staining revealed that the expression for both hBD-1 and SLPI was strongest in the glandular epithelium and areas of high-grade CIN when compared to normal tissue within the same sections. For two women that required a second treatment, the scarred epithelium of the repeat cone exhibited weak staining for both peptides compared to the previously untreated epithelium (Additional file 1: Figure S3).

### Proinflammatory cytokine levels

**Fig. 6:**
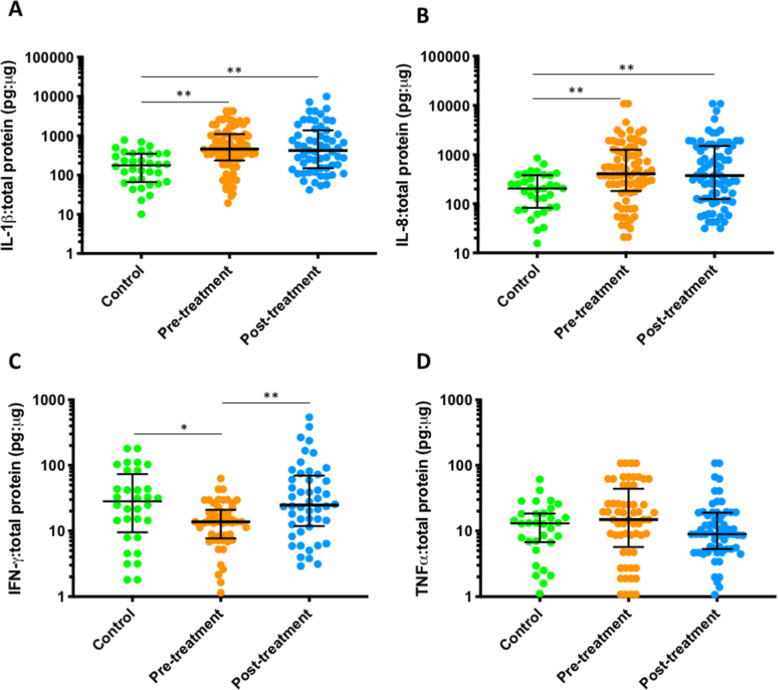
Levels of IL-1β (**A**), IL-8 (**B**), IFN-γ (**C**) and TNF-α (**D**) in 80 treated women and 34 controls. IL-1β (**A**) and IL-8 (**B**) were both significantly elevated prior to treatment in 80 women compared to 34 healthy controls (*p* < 0.0001 and *p* = 0.0014, respectively, unpaired *t* test). There was no significant change in the levels after excision, and they remained significantly higher than healthy controls (*p* < 0.0001, unpaired *t* test and *p* = 0.0035, paired *t* test, respectively). IFN-γ (**C**) was significantly lower prior to treatment compared to controls (*p* = 0.01, unpaired *t* test). Levels were significantly higher after treatment (*p* = 0.002, paired *t* test) and were similar to those seen in controls (*p* > 0.99, unpaired *t* test). TNF-α (**D**) was raised pre-treatment compared to controls, but this was not significant (*p* = 0.62, unpaired *t* test). After treatment, TNF-α levels fell when compared to the paired pre-treatment levels but this was non-significant (*p* = 0.10, paired *t* test) and was found at a level similar to that of untreated controls (*p* > 0.99, unpaired *t* test). Dots depict individual samples; error bars denote median and interquartile ranges, ***p < 0.001; **p < 0.01; *p < 0.05

The levels of proinflammatory cytokines were measured in 80 treated women and 34 healthy controls and normalised to levels of total protein in each sample (Additional file 1: Table S5, Fig. 6). Only four out of the 10 cytokines had detectable levels. The proinflammatory cytokines IL-1β and IL-8 were both significantly elevated prior to treatment compared to healthy controls (*p* < 0.0001 and *p* = 0.0014, respectively, unpaired *t* test), did not change after excision and maintained significantly higher levels post-treatment than controls (*p* < 0.0001 and *p* = 0.0035, respectively, unpaired *t* test). The length of excision and regeneration did not impact concentration (data not shown). The same patterns were seen for sub-analyses 3A and 3B with IL-1β and IL-8 both being higher pre-treatment compared to controls and remaining elevated after treatment. TNF-α was also elevated prior to treatment compared to controls, but this was not significant (*p* = 0.62, unpaired *t* test), and after treatment, levels fell although this was not significant (*p* = 0.10, paired *t* test) when compared to pre-treatment levels. The levels of TNF-α post-treatment were similar to those seen in untreated controls (*p* > 0.99, unpaired *t* test). IFN-γ was significantly lower prior to treatment compared to controls (*p* = 0.01, unpaired *t* test), whilst levels after treatment were significantly higher than before (*p* = 0.002, paired *t* test) and similar to levels seen in healthy controls. IL-2, IL-4, IL-6, IL-10, MIP-1α and RANTES were below the lower limit of quantification in the majority of samples.

### Correlation between bacterial species, cytokines and antimicrobial peptides

**Fig. 7:**
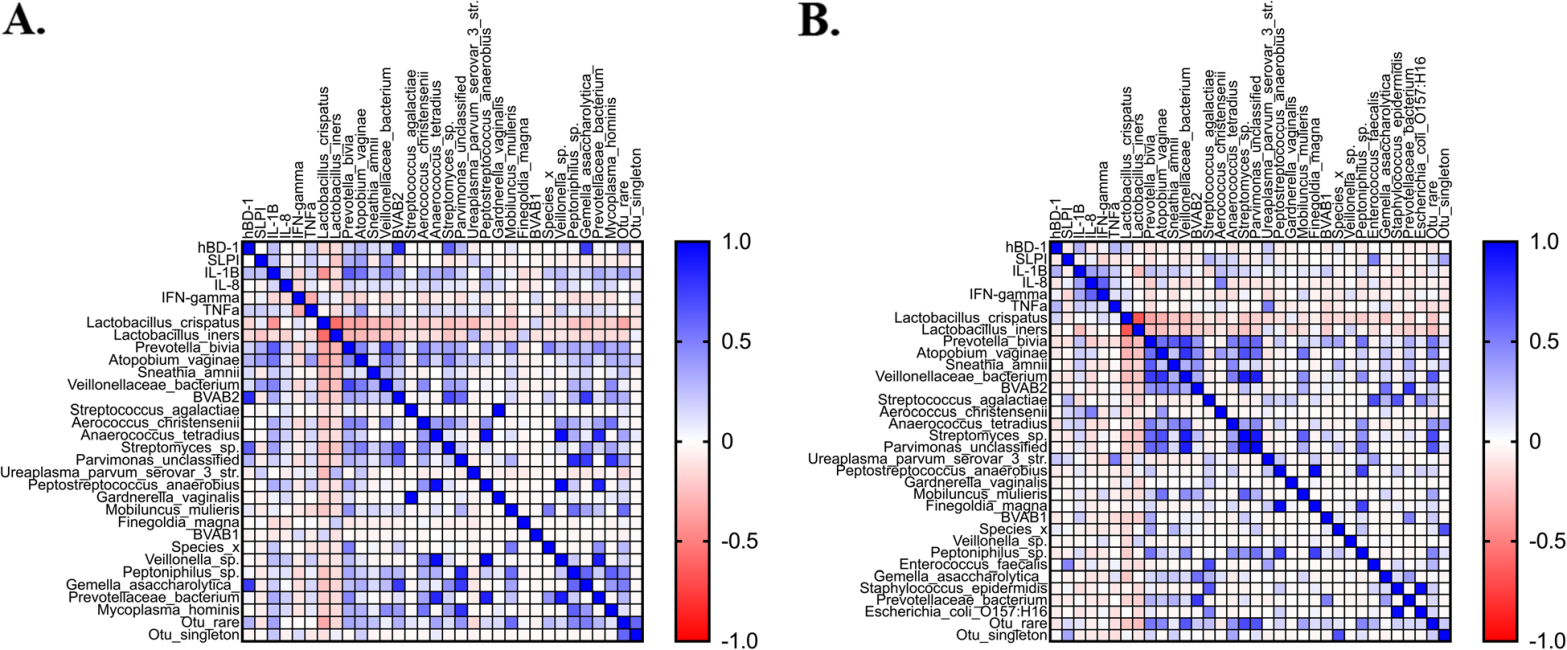
Correlation between bacterial species, antimicrobial peptides and cytokines before (**A**) and after (**B**) treatment in 56 treated women who subsequently had normal cytology, HPV negative at 6-month follow-up (Pearson correlation coefficient). Anaerobes such as *Prevotella bivia* (*p* = 0.02, Pearson’s correlation coefficient), *Atopobium vaginae* (*p* = 0.005, Pearson’s correlation coefficient), *BVAB2* (*p* < 0.001, Pearson’s correlation coefficient), *Streptomyces* sp. (*p* < 0.001, Pearson’s correlation coefficient), and *Gemella asaccharolytic* (*p* < 0.001, Pearson’s correlation coefficient) were positively correlated with AMP expression prior to treatment, all of which were also positively correlated with expression of IL-1β (*p* < 0.001, Pearson’s correlation coefficient). After treatment, there is no significant change in bacterial composition, but AMP expression is no longer correlated with these bacterial species

Correlation analysis was performed to further understand the interplay between the bacterial species present and the expression of cytokines and antimicrobial peptides. We found that anaerobic species were positively correlated with the expression of antimicrobial peptides and IL-1β (Additional file 1: Figure S4). In the subgroup analysis comparing only to women that were HPV and cytology-negative at follow-up (56 women), we found even stronger correlations. Anaerobes such as *Prevotella bivia* (*p* = 0.02, Pearson’s correlation coefficient), *Atopobium vaginae* (*p* = 0.005, Pearson’s correlation coefficient), *BVAB2* (*p* < 0.001, Pearson’s correlation coefficient), *Streptomyces* sp. (*p* < 0.001, Pearson’s correlation coefficient) and *Gemella asaccharolytic* (*p* < 0.001, Pearson’s correlation coefficient) were most positively correlated with AMP expression prior to treatment, all of which were also positively correlated with the expression of IL-1β (*p* < 0.001, Pearson’s correlation coefficient). After treatment, there is no significant change in bacterial composition, but AMP expression is no longer correlated with these bacterial species (Fig. 7).

## Discussion

This prospective observational study reports how local excision of CIN affects the vaginal microbiota composition, innate immune and inflammatory response and how the findings pre- and post-treatment compare to healthy untreated controls.

Our findings suggest that women planned for CIN treatment had significantly higher levels of a high diversity, *Lactobacillus* sp.-depleted microbiome pre-treatment compared to a healthy, untreated control group with normal cytology. This finding is in line with previously published cross-sectional data describing associations between the VMB and the presence of CIN [4, 8, 14, 15] with *Atopobium*, *Sneathia* and *Prevotella* species being consistently associated with a higher-grade disease across studies [4, 6, 8, 14, 42], the latter two of which were significantly overrepresented in our pre-treatment cohort compared to controls*.* Our analysis further revealed that excision of CIN does not impact on the VMB composition six months after treatment, which remained more diverse with a decreased relative abundance of *Lactobacillus crispatus* compared to normal controls. The subgroup analyses restricted to HPV-negative women post-treatment, and controls also revealed consistent results. These results contrast with two recently published small studies. Zhang and co-workers reported a shift towards greater *Lactobacillus* sp. abundance at 3-month follow-up in a group of 26 Chinese women, six of whom were post-menopausal women [43]. Wiik and colleagues also reported a shift towards a less diverse VMB in 89 treated Norwegian women [44], although this study used targeted PCR to characterise bacterial species rather than next-generation sequencing (NGS) techniques as used here. Our study is the largest cohort to date of over 100 prospectively collected paired samples in a less diverse population of solely premenopausal women [45].

Whilst overall VMB community structure was no different after treatment within the paired samples from women pre- and post-treatment, we did observe a change in specific bacterial taxa when comparing the pre- and post-treatment samples to untreated, healthy controls. Most notably, *Sneathia amnii* that was overrepresented before treatment when compared to controls was no longer overexpressed after treatment when compared again to controls. Whilst there was a trend for the mean abundance of *Sneathia amnii* to fall after treatment, this was not statistically significant. This suggests that overgrowth of *Sneathia amnii* in women HPV and CIN arises as a consequence of the disease. *Sneathia* species are often seen in women with bacterial vaginosis [46]. There remains a relatively lack of longitudinal studies examining the temporal association with *Sneathia*, HPV and cervical abnormalities. The mechanism by which disease leads to overgrowth of *Sneathia* has not been previously studied. However, *Sneathia* has been consistently suggested to be a microbiological marker of HPV infection [13, 47] and high-grade disease states [4, 8, 42]. Łaniewski and coworkers demonstrated an increasing association with *Sneathia* enrichment in HPV-positive controls, following those with low-grade and then high-grade lesions, compared to HPV-negative controls [8], which further suggests the bacteria appears to thrive as the result of an HPV-diseased cervix. Researchers have explored how *Sneathia* may promote carcinogenesis. Sequencing of *Sneathia*’s genome has revealed the genes involved in cervical cell adhesion, cytotoxicity and epithelial cell damage [48]. Liquid chromatography mass spectroscopy has revealed metabolites related to nucleotide biosynthesis, amino acid catabolism and mucosal inflammation [20]. Furthermore, co-culture with ME-180 human cervical cells demonstrated epithelial cell perforation close to the sites of *Sneathia* adhesion [48], which is a necessary step to facilitate entry of HPV to cervical cells.

In spite of a reduction in *Sneathia*, other bacterial vaginosis-associated bacteria including *Prevotella bivia* were persistently elevated both before and after treatment compared to controls, with a relative lack of *Lactobacillus* spp. *Lactobacillus* spp. are able to outcompete the growth of *Gardnerella* and *Prevotella* species [49] and has been shown to inhibit the growth of cervical cancer cell lines [50]. Further in vitro studies have demonstrated that *L. crispatus* in particular is protective against inflammation-mediated increases in ectocervical cell permeability, as the endocervical production of IL-6 and IL-8 significantly decreased after exposure to *L. crispatus* bacteria-free supernatants [51]. *Prevotella bivia* has been suggested as an early coloniser in BV and high-diversity models involved in biofilm formation and subsequently paves the way for secondary colonisers such as *Atopobium* and *Sneathia* species [52]. This presents an entirely plausible mechanism for an increased risk of subsequent HPV infection, CIN recurrence and cervical cancer development.

One of the major strengths of this study is the inclusion of paired samples and the further comparisons to an untreated control population with normal cytology that permitted us to further infer on the causal relationship between the VMB and cervical disease. A number of cross-sectional studies of HPV positive [13, 53, 54] and high-grade CIN cohorts [4, 14, 15], have proposed that increased bacterial diversity in these women is a consequence of the pathology state. However, our results showing no change in VMB composition following excision of CIN leads us to conclude that the presence of viral infection and cervical disease does not drive high diversity [3, 5]. Rather, it appears more plausible that women with CIN have inherent genetic, epigenetic and environmental factors that shape VMB composition before and after treatment. The presence of this ongoing high-diversity VMB may induce a proinflammatory response as reflected by the high levels of proinflammatory cytokines post-treatment, most notably IL-1β and IL-8 which remained persistently elevated after treatment compared to controls despite the successful removal of the disease. This may be a factor that predisposes these women to the acquisition of oncogenic HPV infections, persistence and ultimately cervical oncogenesis. Consistent with this notion, clinical epidemiological studies have consistently reported higher CIN recurrence rates and increased incidence of invasive cervical and other HPV-related cancers in women treated with conisation for CIN [55–57]. Although this increased incidence may be partly explained by residual infection/disease [56] and/or poor compliance with follow-up [55], it is also possible that these high-risk women are highly sensitive to infection by HPV and as a result are more susceptible to the development of persistent infection, precancer and ultimately cancer. Although a number of viral or host-related genetic, epigenetic and immunological factors may be responsible for this susceptibility, it is also possible that an inherent genetic or environmentally driven VMB diversity and the associated inflammation create a pro-carcinogenic environment that permits HPV to express its oncogenic potential [5].

The factors that dictate a woman’s vaginal microbiota structure are not well understood. Whilst environmental factors, such as smoking, endogenous hormone use and hygiene practices [58–60] are known to alter VMB composition, genetic factors have also been described based on twin cohort studies [13, 61]. Genetic polymorphisms have been reported in women with recurrent bacterial vaginosis in a variety of genes including those encoding mannose-binding lectin [62, 63] and IL-1β [64], both of which play key immune roles.

Our results permit further exploration of inflammatory mechanisms that promotes disease progression [65–67]. There has been an ongoing effort to understand the mechanisms interlinking the VMB with mucosal immune response. The consensus amongst a series of small studies is that *Lactobacillus* spp. depleted, high diversity VMB induces over-expression of pro-carcinogenic biomarkers and cyto/chemokines leading to an altered mucosal immune proinflammatory environment [8, 9, 20, 42]. In our cohort, the high levels of IL-1β and IL-8 pre-treatment, previously shown to be elevated in women with CIN [18], remained significantly higher after treatment suggesting ongoing mucosal inflammation even in the absence of disease. In contrast, high TNF-α levels of pre-treatment fell and became similar to the levels seen in the control group, suggesting that this cytokine overexpression is induced by the disease and is reversed when the diseased tissue is excised in line with previous reports associating elevated TNF-α levels to HPV persistence and disease progression [19]. The expression of INFγ, an antiviral cytokine is known to be supressed by HPV oncogenes E6 and E7 in women with high-grade disease [21, 68]. In our cohort, the low levels of pre-treatment became similar to those of controls after treatment. Our results are in agreement with a study by Saftlas and colleagues of 65 treated women and 76 controls, reporting similar trends for cytokine levels although their results were largely non-significant [69].

The cervix plays an immunological role in protecting against bacterial and viral species, and its production of antimicrobial peptides (AMPs) is one layer in this sophisticated mucosal defence. Human beta-defensin-1 is an AMP with broad-spectrum antibacterial and antiviral activity [70, 71], and secretory leucocyte protease inhibitor (SLPI) which has been found at high concentration in the mucus plug likely plays a role in protection from infectious agents in pregnancy [72] and is upregulated in cervical cancer cases [27]. Furthermore, both are suggested to play a role in defence against HPV [26, 73]. Polymorphisms in the DEFB1 gene that encodes hBD-1 have been associated with increased susceptibility to HPV infection [24], and upregulated hBD-1 expression has been observed in low-risk HPV-mediated genital warts [74]. SLPI has potent vial activity against HIV [75], HSV [76], HPV (in head and neck cancers) [25] and upregulation is seen in CIN [26] and cervical cancer [27].

The exploration of the two antimicrobial peptides hBD1 and SLPI known to play a role in mucosal defence was significantly lower after treatment compared to untreated controls. Although these were heavily expressed in areas of high-grade disease versus normal tissue within the same sections, these were largely deficient in the healed scar on the treated cervix in two women that had repeat cones and those sections of these cones were compared by immunohistochemistry. It was not therefore surprising that excision of CIN dropped AMP levels, as the disease is likely to drive their expression. Most strikingly, however, AMP levels remained significantly lower after treatment when compared to controls; both groups were free of disease. Correlation analysis showed that particular anaerobic species are positively correlated with AMP expression prior to treatment. Although the bacterial composition remains the same after treatment, these species do not appear to induce the same expression of AMPs in the scarred cervix. These low AMP levels that do not respond to the bacterial species and persistent increased inflammation after treatment further support an inferior functional immune deficit of the treated cervix which may contribute to the increased risk of adverse obstetric outcomes and disease recurrence in these treated women [40, 77–79].

There were of course limitations. Although this is the largest conducted study with over 100 paired samples from treated women and the first to include an untreated healthy control population, the sample size in different subgroup comparisons was small. We only collected samples at baseline and 6-month follow-up, in line with current routine visits in the clinic. More intensive sampling of the patients after excision may provide further information on the transition between different community state types and immune states post-treatment. With regard to the sequencing, counts were normalised to 296 reads, and whilst this appears low, we were able to retain 99.7% of OTU counts and still provided coverage of > 97.9% for all samples. Finally, our predominantly young control group recruited in a central London hospital had a high HPV positivity rate of 31%. Although this is higher than the overall rate reported by Rebolj and co-workers in 578,547 women attending the English Cervical Screening Programme, it is in line with the 28% HPV positivity in 24–29-year-olds reported by the age-stratified data in the same cohort [80].

## Conclusions

This prospective observational study represents the largest available cohort of paired samples investigating the impact of excision of CIN on the VMB composition, along with immune factors, and is the first to compare these to an untreated control group. This is the first study to report that women with CIN have a VMB composition that remains unchanged despite the removal of the disease suggesting that there be other genetic or environmental factors that determine a woman’s vaginal microbiota structure. This permits further inference to causality and hints that the high diversity of the microbiota in women with CIN is a predisposing factor to the acquisition of HPV and development of precancer rather than being caused by CIN itself. This inherent high rate of high diversity microbiome in these women coupled with a proinflammatory state as a result as shown by the cytokine levels may predispose these women to persistent HPV infection, progressive disease and ultimately malignant transformation. This further supports the concept that manipulation of the VMB with pre- and probiotics or even vaginal microbiota transplants [81] may represent a novel therapeutic target.

## Supplementary Information

**Additional file 1:.** The Vaginal Microbiota and Innate Immunity After Local Excisional Treatment for Cervical Intraepithelial Neoplasia, Supplementary tables (Table S1 – S5) and figures (Figure S1 – S4).

## Data Availability

The datasets generated and/or analysed during the current study are available in the European Nucleotide Archive’s (ENA) Sequence Read Archive (SRA) (https://www.ebi.ac.uk/ena/browser/view/PRJEB40437) [82].
