# Supplementary material for: The vaginal microbiota and innate immunity after local excisional treatment for cervical intraepithelial neoplasia
Source: Genome Med. 2021 Nov 4;13:176. doi: 10.1186/s13073-021-00977-w (PMC8567681; doi:10.1186/s13073-021-00977-w)
Supplement: Supplementary file 1 — Additional file 1:. The Vaginal Microbiota and Innate Immunity After Local Excisional Treatment for Cervical Intraepithelial Neoplasia, Supplementary tables (Table S1 – S5) and figures (Figure S1 – S4). [file 13073_2021_977_MOESM1_ESM.docx]

Table S1. Patient characteristics for Sub-group analyses

|  | **Sub-group Analysis 3A** | | | **Sub-group Analysis 3B** | | |
| --- | --- | --- | --- | --- | --- | --- |
|  | **Post-treatment, normal cyto, n=81** | **Control,**  **n=39** | **p value** | **Post-treatment, Normal cyto, HPV negative,**  **n=70** | **Control,**  **HPV negative,**  **n=23** | **p value** |
| **Age, years** |  |  | 0.1144 |  |  | **0.0106** |
| Mean (SD, range) | 31.6 (5.1, 25 – 43) | 30.1 (4.4, 20 – 43) |  | 31.7 (5.2, 25 – 43) | 29.8 (3.3, 24 – 37) |  |
| **Ethnicity , n/N (%)** |  |  | 0.2098 |  |  | **0.0487** |
| Caucasian | 69/81 (86) | 30/39 (77) |  | 59/70 (84) | 15/23 (65) |  |
| Asian | 6/81 (7) | 2/39 (5) |  | 6/70 (9) | 2/23 (9) |  |
| Black | 6/81 (7) | 7/39 (18) |  | 5/70 (7) | 6/23 (26) |  |
| **Parity, n/N (%)** |  |  | 0.5168 |  |  | 0.2848 |
| Nulliparous | 56/81 (69) | 30/39 (76) |  | 48/70 (69) | 19/23 (80) |  |
| Parous | 25/81 (31) | 9/39 (24) |  | 22/70 (31) | 4/23 (20) |  |
| **Smoking status, n/N (%)** |  |  | 0.3447 |  |  | 0.1413 |
| Current smoker | 20/81 (25) | 6/39 (15) |  | 18/70 (25) | 2/23 (9) |  |
| Non-smoker | 61/81 (75) | 33/39 (85) |  | 52/70 (75) | 20/23 (91) |  |
| **Phase of menstrual cycle, n/N (%)** |  |  | 0.3257 |  |  | 0.5137 |
| Luteal | 36/81 (45) | 23/39 (58) |  | 31/70 (44) | 13/23 (56) |  |
| Follicular | 40/81 (49) | 14/39 (36) |  | 34/70 (49) | 8/23 (35) |  |
| Unknown | 5/81 (6) | 2/39 (5) |  | 5/70 (7) | 2/23 (9) |  |
| **Contraception, n/N (%)** |  |  | 0.8009 |  |  | 0.4619 |
| Nil | 28/81 (35) | 19/39 (49) |  | 24/70 (35) | 12/23 (55) |  |
| Condoms | 16/81 (20) | 5/39 (13) |  | 15/70 (21) | 2/23 (9) |  |
| COCP | 27/81 (33) | 12/39 (31) |  | 22/70 (32) | 7/23 (32) |  |
| POP | 3/81 (4) | 1/39 (2) |  | 3/70 (4) | 1/23 (4) |  |
| Copper IUD | 1/81 (1) | 0/39 (0) |  | 1/70 (1) | 0/23 (0) |  |
| Mirena IUS | 4/81 (5) | 1/39 (2) |  | 3/70 (4) | 0/23 (0) |  |
| Contraceptive implant | 2/81 (2) | 1/39 (2) |  | 2/70 (3) | 0/23 (0) |  |
| **Time since last intercourse, n/N (%)** |  |  | 0.3321 |  |  | 0.7044 |
| >48 hours | 72/81 (90) | 34/39 (87) |  | 63/70 (90) | 20/23 (87) |  |
| <48 hours | 8/81 (10) | 5/39 (13) |  | 7/70 (10) | 3/23 (13) |  |
| **HPV status** |  |  | **0.0006** |  |  | >0.9999 |
| Negative | 70/81 (86) | 23/39 (59) |  | 70/70 (100) | 23/23 (100) |  |
| Positive | 11/81 (14) | 12/39 (31) |  | 0/70 (0) | 0/23 (0) |  |
| Unknown | 0/81 (0) | 4/39 (10) |  | 0/70 (0) | 0/23 (0) |  |
| **Cytology** |  |  | >0.9999 |  |  | >0.9999 |
| Negative | 81/81 (100) | 39/39 (100) |  | 70/70 (100) | 23/23 (100) |  |
| BNC | 0/81 (0) | 0/39 (0) |  | 0/70 (0) | 0/23 (0) |  |
| LSIL | 0/81 (0) | 0/39 (0) |  | 0/70 (0) | 0/23 (0) |  |
| HSIL | 0/81 (0) | 0/39 (0) |  | 0/70 (0) | 0/23 (0) |  |
| **Cytology & HPV status** |  |  | **0.0006** |  |  | >0.9999 |
| Normal, HPV -ve | 70/81 (86) | 23/39 (59) |  | 70/70 (100) | 23/23 (100) |  |
| Normal, HPV +ve | 11/81 (14) | 12/39 (31) |  | 0/70 (0) | 0/23 (0) |  |
| Normal, HPV status unknown | 0/81 (0) | 4/39 (10) |  | 0/70 (0) | 0/23 (0) |  |
| BNC, HPV -ve | 0/81 (0) | 0/39 (0) |  | 0/70 (0) | 0/23 (0) |  |
| BNC, HPV +ve | 0/81 (0) | 0/39 (0) |  | 0/70 (0) | 0/23 (0) |  |
| LSIL, HPV -ve | 0/81 (0) | 0/39 (0) |  | 0/70 (0) | 0/23 (0) |  |
| LSIL, HPV +ve | 0/81 (0) | 0/39 (0) |  | 0/70 (0) | 0/23 (0) |  |
| HSIL | 0/81 (0) | 0/39 (0) |  | 0/70 (0) | 0/23 (0) |  |

*BNC: Borderline nuclear changes; CIN: Cervical intraepithelial neoplasia; COCP: Combined oral contraceptive pill; HSIL: high-grade squamous intraepithelial neoplasia; HPV: Human Papillomavirus; IUD: Intrauterine device; IUS: Intrauterine system; LSIL: low-grade squamous intraepithelial neoplasia; POP: Progesterone-only pill, SD: standard deviation.*

**Table S2. Pre- and post-treatment CST and VMB dynamics according to cone length, proportion of cervical length excised and proportion of cervical length regeneration.**

|  |  | **Cone length(mm)**  Median 11.0 mm, range 7.0-22.0mm | | | **Proportion of cervical length excised (%)**  Median 33.2 %, range 22.0-79.6% | | | **Proportion of cervical length regenerated (%)**  Median 73.5 %, range 12.0-98.5% | | |
| --- | --- | --- | --- | --- | --- | --- | --- | --- | --- | --- |
|  |  | **Lower 50^th^ percentile,**  **n=55** | **Upper 50^th^ percentile,**  **n=48** | **p value** | **Lower 50^th^ percentile,**  **n=52** | **Upper 50^th^ percentile,**  **n=51** | **p value** | **Lower 50^th^ percentile,**  **n=52** | **Upper 50^th^ percentile,**  **n=51** | **p value** |
| **Pre-treatment** | **CST I**  (*L. crispatus*) | 26/55 (47) | 19/48 (40) | 0.7375 | 24/52 (46) | 21/51 (41) | 0.2091 | 22/52 (42) | 23/51 (45) | 0.6000 |
|  | **CST III**  (*L. iners*) | 18/55 (33) | 19/48 (40) |  | 21/52 (40) | 16/51 (31) |  | 21/52 (40) | 16/51 (31) |  |
|  | **CST IV** (*Lactobacillus* spp. deplete, high diversity) | 11/55 (20) | 10/48 (20) |  | 7/52 (14) | 14/51 (28) |  | 9/52 (18) | 12/51 (24) |  |
| **Post-treatment** | **CST I**  (*L. crispatus*) | 24/55 (44) | 16/48 (34) | 0.5750 | 20/52 (38) | 20/51 (39) | 0.9999 | 23/52 (44) | 17/51 (33) | 0.3527 |
|  | **CST III**  (*L. iners*) | 22/55 (40) | 22/48 (46) |  | 22/52 (42) | 22/51 (43) |  | 22/52 (42) | 22/51 (43) |  |
|  | **CST IV** (*Lactobacillus* spp. deplete, high diversity) | 9/55 (16) | 10/48 (20) |  | 10/52 (20) | 9/51 (18) |  | 7/52 (14) | 12/51 (24) |  |
| **Dynamics of CST’s** | **Same CST pre & post treatment** | 36/55 (65) | 22/48 (46) | **0.0498** | 31/52 (60) | 27/51 (53) | 0.5539 | 31/52 (60) | 27/51 (53) | 0.5539 |
|  | **Changed CST pre & post treatment** | 19/55 (35) | 26/48 (54) |  | 21/52 (40) | 24/51 (47) |  | 21/52 (40) | 24/51 (47) |  |
|  | **Changed from one *Lactobacillus* dominant CST to another** | 5/19 (26) | 9/26 (35) | 0.7460 | 12/21 (57) | 13/24 (54) | 1.000 | 13/21 (62) | 12/24 (50) | 1.000 |
|  | **Changed from *Lactobacillus* dominant CST to CST IV** | 3/19 (16) | 6/26 (23) | 0.7123 | 3/21 (14) | 6/24 (25) | 0.4764 | 3/21 (14) | 6/24 (25) | 0.4764 |
|  | **Changed from CST IV to a *Lactobacillus* dominant CST** | 5/19 (26) | 6/26 (23) | 1.000 | 3/21 (14) | 8/24 (33) | 0.1772 | 0/21 (0) | 1/24 (4) | 1.000 |

*CST: Community state type, L.: Lactobacillus*

Table S3. Cervical length regeneration according to pre- and post-treatment CST

| **Regeneration according to pre-treatment CST** | | | | |
| --- | --- | --- | --- | --- |
|  | **Length of cone excised, mm**  Average  (range, SD) | **Cervical length at follow-up, mm**  Average  (range, SD) | **Proportional cervical length excised, %**  Average  (range, SD) | **Proportion cervical length regeneration, %**  Average  (range, SD) |
| **CST I,**  n = 45 | 11.4  (7 – 22, 3.3) | 29.5  (23 – 42, 6.9) | 33.4  (12 – 80, 12.6) | 62.1  (14 – 98, 24.9) |
| **CST III,**  n = 37 | 12.0  (7 – 21, 3.6) | 28.4  (24 – 39, 6.7) | 36.8  (18 – 62, 11.0) | 70.4  (12 – 97, 21.3) |
| **CST IV,**  n = 21 | 11.6  (7 – 18, 3.8) | 30.1  (22 – 36, 4.0) | 32.4  (11 – 52, 11.6) | 70.7  (12 – 98, 22.4) |
| **p value** | 0.8490 | 0.8253 | 0.7027 | 0.3828 |
| **Regeneration according to post-treatment CST** | | | | |
|  | **Length of cone excised, mm**  Average  (range, SD) | **Cervical length at follow-up, mm**  Average  (range, SD) | **Proportional length excised, %**  Average  (range, SD) | **Proportion length regeneration, %**  Average  (range, SD) |
| **CST I,**  n = 41 | 10.8  (7 – 21, 3.6) | 30.0  (24 – 42, 8.1) | 31.8  (12 – 62, 11.8) | 67.5  (15 – 94, 22.1) |
| **CST III,**  n = 39 | 12.1  (7 – 17, 2.8) | 29.2  (22 – 39, 4.8) | 35,8  (18 – 55, 9.0) | 68,0  (12 – 97, 24.9) |
| **CST IV,**  n = 16 | 12.2  (7 – 18, 3.8) | 28.2  (22 – 36, 4.5) | 37.7  (11 – 56, 13.0) | 62.5  (12 – 98, 23.0) |
| **p value** | 0.3418 | 0.8679 | 0.3301 | 0.6048 |

*CST: Community state type, SD: standard deviation.*

Table S4. hBD-1 and SLPI levels per total protein concentration

|  | **hBD-1:total protein (pg/μg protein)**  (median ± IQR) | **SLPI:total protein (pg/μg protein)**  (median ± IQR) |
| --- | --- | --- |
| **Analysis 1** |  |  |
| Pre-treatment, n=80 | 5651 ± 6284 | 221437 ± 421810 |
| Control, n=34 | 2096 ± 3503 | 86673 ± 97675 |
| p value | **0.0033** | **0.0006** |
| **Analysis 2** |  |  |
| Pre-treatment, n=80 | 5651 ± 6284 | 221437 ± 421810 |
| Post-treatment, n=80 | 1007 ± 423 | 28392 ± 89230 |
| p value | **<0.0001** | **<0.0001** |
| **Analysis 3** |  |  |
| Post-treatment, n=80 | 1007 ± 1552 | 28392 ± 89230 |
| Control, n=34 | 2096 ± 3503 | 86673 ± 97675 |
| p value | **0.0029** | **0.0382** |
| **Sub-group analysis 3A** |  |  |
| Post-treatment, normal cytology, n=65 | 956 ± 1388 | 36825 ± 70571 |
| Control, n=34 | 2096 ± 3503 | 86673 ± 97675 |
| p value | **0.0003** | **0.0009** |
| **Sub-group analysis 3B** |  |  |
| Post-treatment, Normal cyto, HPV  negative, n=50 | 1038 ± 1523 | 26298 ± 32916 |
| Control, HPV negative, n=20 | 2625 ± 5042 | 81669 ± 94238 |
| p value | **0.0006** | **0.0016** |

*HPV: Human Papillomavirus;* IQR; interquartile range

Table S5. Impact of treatment on levels of Levels of IL-1β, IL-8, IFN-γ and TNF-α

|  | **IL-1β:total protein (pg/μg protein)**  (median ± IQR) | **IL-8:total protein (pg/μg protein)**  (median ± IQR) | **Interferon-γ**  **pg/μg protein)**  (median ± IQR) | **TNF-α:total protein (pg/μg protein)**  (median ± IQR) |
| --- | --- | --- | --- | --- |
| **Analysis 1** |  |  |  |  |
| Pre-treatment, n=80 | 463.9 ± 714.2 | 408.8 ± 1085.2 | 13.7 ± 13.3 | 14.9 ± 39.3 |
| Control, n=34 | 177.4 ± 284.3 | 199.9 ± 309.5 | 28.2 ± 74.5 | 13.1 ± 11.4 |
| p value | **<0.0001** | **0.0014** | **0.01** | 0.62 |
| **Analysis 2** |  |  |  |  |
| Pre-treatment, n=80 | 463.9 ± 714.2 | 408.8 ± 1085.2 | 13.7 ± 13.3 | 14.9 ± 39.3 |
| Post-treatment, n=80 | 497.8 ± 666.1 | 374.4 ± 1302.3 | 24.7 ± 58.2 | 8.9 ± 13.7 |
| p value | >0.99 | >0.99 | **0.002** | 0.10 |
| **Analysis 3** |  |  |  |  |
| Post-treatment, n=80 | 497.8 ± 666.1 | 374.4 ± 1302.3 | 24.7 ± 58.2 | 8.9 ± 13.7 |
| Control, n=34 | 177.4 ± 284.3 | 199.9 ± 309.5 | 28.2 ± 74.5 | 13.1 ± 11.4 |
| p value | **<0.0001** | **0.0035** | >0.99 | >0.99 |
| **Sub-group analysis 3A** |  |  |  |  |
| Post-treatment, normal cytology, n=65 | 421.7 ± 614.3 | 425.7 ± 1378.3 | 23.7 ± 54.6 | 9.6 ± 13.7 |
| Control, n=34 | 177.4 ± 284.3 | 199.9 ± 309.5 | 28.2 ± 74.5 | 13.1 ± 11.4 |
| p value | **0.0002** | **0.0005** | 0.4408 | 0.7102 |
| **Sub-group analysis 3B** |  |  |  |  |
| Post-treatment, Normal cyto, HPV  negative, n=50 | 406.7± 639.7 | 362.6 ± 1358.0 | 23.8 ± 51.5 | 8.9 ± 9.6 |
| Control, HPV negative, n=20 | 264.6 ± 343.2 | 116.6 ± 314.0 | 32.15 ± 58.8 | 13.1 ± 14.9 |
| p value | **0.0097** | **0.0023** | 0.3721 | 0.3156 |

*HPV: Human Papillomavirus;* IQR; interquartile range

**SUPPLEMENTARY FIGURES**

**A. Normal controls VS Post-treatment normal cytology (Subgroup Analysis 3A)**

**i. ii.**


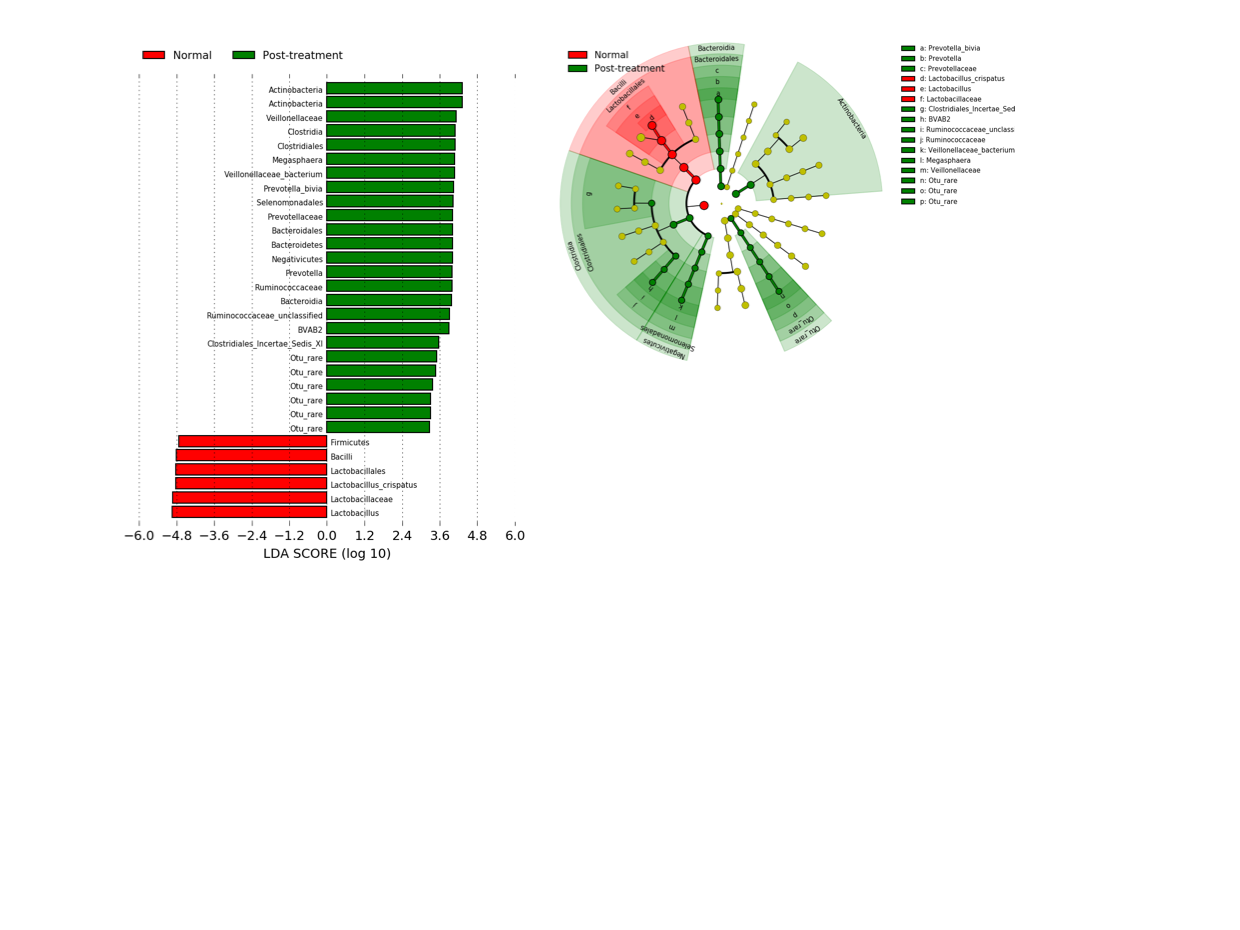


**B. Normal HPV negative controls VS Post-treatment cytology & HPV negative (Subgroup Analysis 3B)**

**i. ii.**


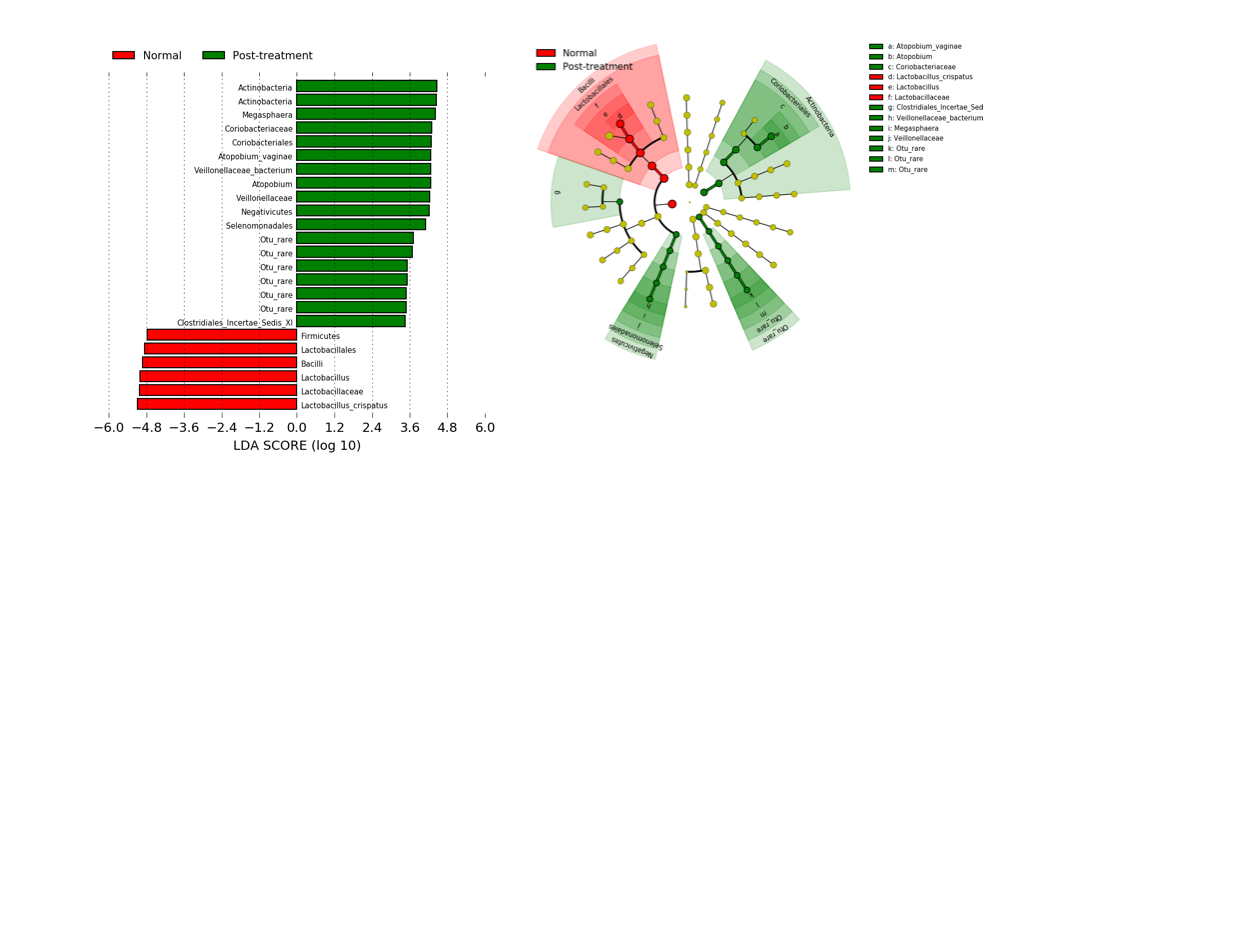


**Figure S1. LEfSe analysis identified vaginal microbiota differentially abundant taxa in the comparison of women with normal cytology post-treatment versus normal controls (Analysis 3A) and negative HPV and cytology post-treatment versus HPV and cytology negative controls (Analysis 3B).**

(A) Analysis 3A: *Prevotella bivia* and BVAB2 were significant overrepresented at a species level in treated women with normal cytology compared to untreated controls. (B) Analysis 3B: *Atopobium vaginae* was overrepresented in the subgroup analysis restricted with HPV-negative treated women and HPV-negative treated controls. *Lactobacillus crispatus* was significantly more abundant in the control groups in both sub-group analyses (A & B).

1. Histogram of LDA scores found to differ significantly in abundance between treated women compared to controls.
2. Cladogram representing taxa at all phylogenetic levels with significantly different abundance in the compared groups. The size of the circle is proportional to the abundance of taxon represented.

*KEY - LDA: linear discriminant analysis. NB. Analysis restricted to top 20 taxa with all remaining taxa denoted as ‘other*

**

**Figure S2. Relative abundance of *Sneathia amnii* before and after treatment.**

There was a decrease in the mean relative abundance of *Sneathia amnii i*n 103 paired samples before and after treatment, with the majority of subjects experiencing a decrease in abundance, however this was not statistically significant (mean pre-treatment 4.36% vs post-treatment 2.51%, p=0.2404, paired t-test).


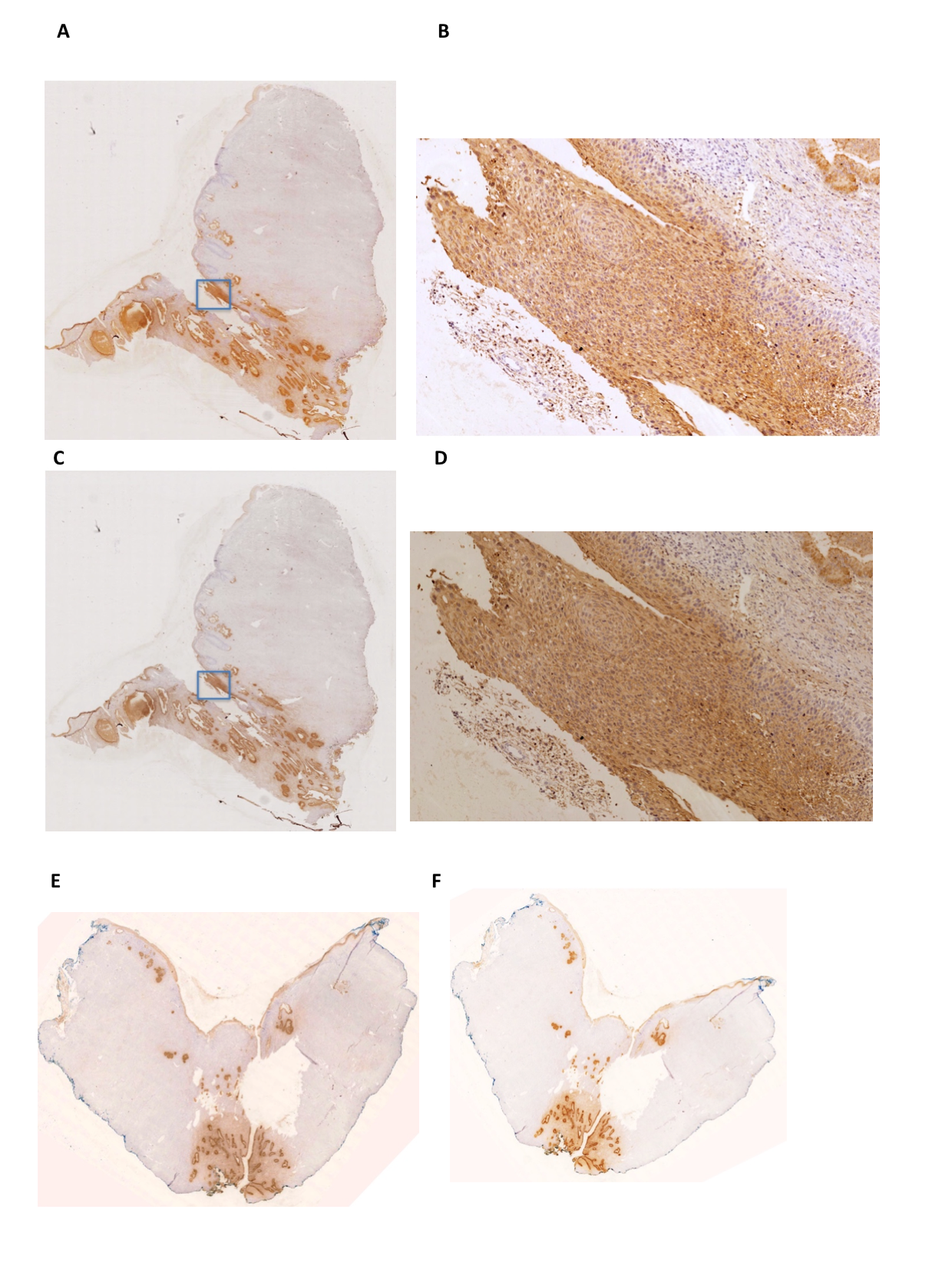


**Figure S3.** **Distribution of hBD1 and SLPI in a section from a cervical cone with CIN3 in the whole section and in an area with CIN3 (hBD1: A – B; SLPI: C – D). Distribution of hBD1 (E) and SLPI (F) after the same women after a second cone.**

Staining for both peptides was strongest in the glandular epithelium and areas of high-grade CIN with weak staining of the normal squamous epithelium. The scarred epithelium appears to exhibit weak staining for both peptides compared to the untouched cervical and glandular epithelial surfaces.

**A. B.**

**
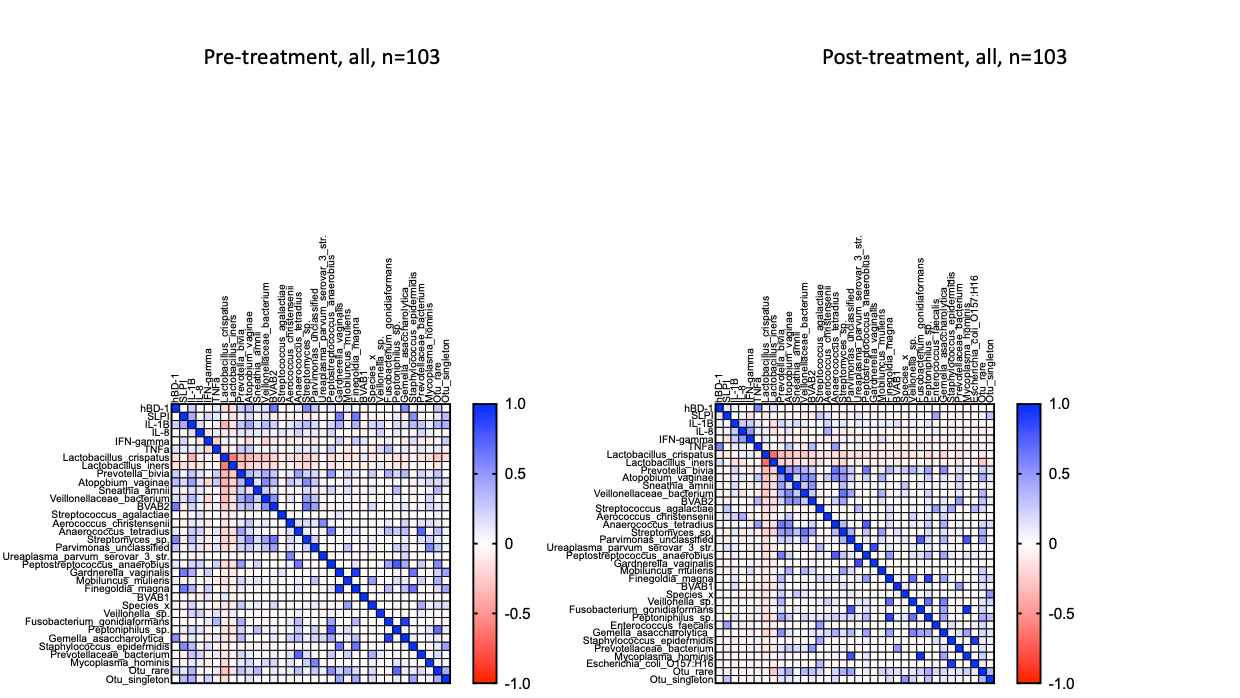
**

**Figure S4. Correlation between bacterial species, antimicrobial peptides and cytokines before (A) and after (B) treatment in 80 treated women (Pearson correlation coefficient).**

Anaerobes such as *Prevotella bivia* (p=0.02)*, Atopobium vaginae* (p=0.003)*, BVAB2* (p<0.001)*, Gardnerella vaginalis* (p<0.001), *Streptomyces sp.* (p<0.001) & *Gemella asaccharolytic* (p<0.001)were positively correlated with AMP expression prior to treatment, all of which were also weakly positively correlated with expression of IL-1β and TNF-α. After treatment there is no significant change in bacterial composition, but AMP expression is no longer correlated with these bacterial species.
